# Correction: Santos et al. The Mitochondrial Antioxidant Sirtuin3 Cooperates with Lipid Metabolism to Safeguard Neurogenesis in Aging and Depression. *Cells* 2022, *11*, 90

**DOI:** 10.3390/cells13151239

**Published:** 2024-07-24

**Authors:** Sónia Sá Santos, João B. Moreira, Márcia Costa, Rui S. Rodrigues, Ana M. Sebastião, Sara Xapelli, Susana Solá

**Affiliations:** 1 Research Institute for Medicines (iMed.ULisboa), Faculty of Pharmacy, Universidade de Lisboa, 1649-003 Lisbon, Portugal; joaomoreira@medicina.ulisboa.pt (J.B.M.); mafi.mafi@gmail.com (M.C.); 2 Instituto de Medicina Molecular (iMM) João Lobo Antunes, Faculdade de Medicina, Universidade de Lisboa, 1649-028 Lisbon, Portugal; rmsrodrigues@medicina.ulisboa.pt (R.S.R.); anaseb@medicina.ulisboa.pt (A.M.S.); sxapelli@medicina.ulisboa.pt (S.X.); 3 Department of Translational Neurodegeneration, German Center for Neurodegenerative Diseases (DZNE), 81377 Munich, Germany; 4 Instituto de Farmacologia e Neurociências, Faculdade de Medicina, Universidade de Lisboa, 1649-028 Lisbon, Portugal

## Error in Figure

In the original article [1], there was a mistake in Figure 4B (upper right panels) as published. By mistake, the representative images of SA-β-gal staining in neural stem cells of the control condition became overlapped with the representative figures of SIRT3 overexpression. The corrected Figure 4B (upper right panels) appears below. The authors state that the scientific conclusions are unaffected. This correction was approved by the Academic Editor. The original publication has also been updated.

## Figures and Tables

**Figure 4:**
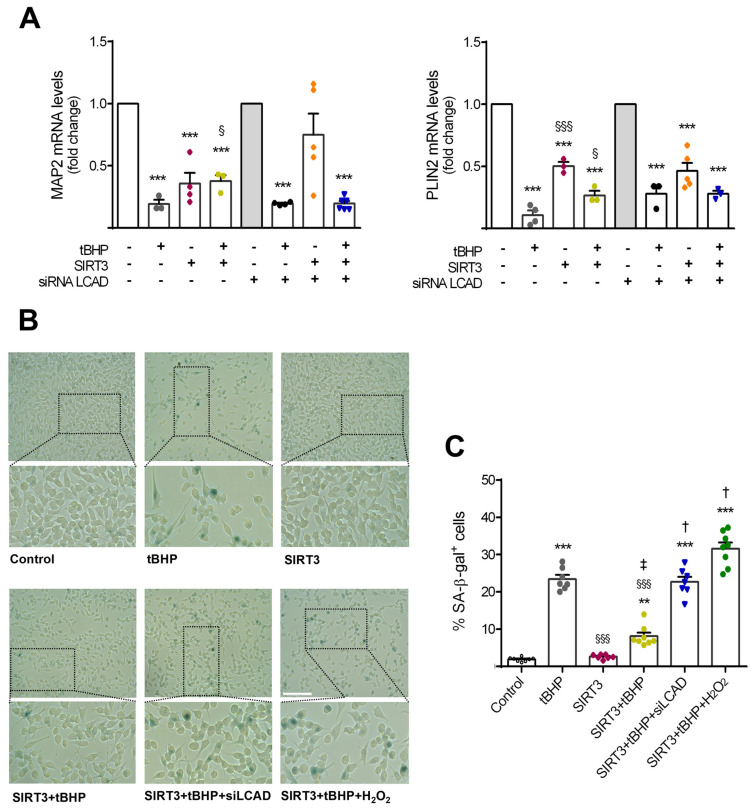
SIRT3 requires LCAD and oxidative control to prevent NSC aging. Mouse NSCs were treated with 50 μM tBHP for 2 h/day, for 4 consecutive days, in self-renewal conditions. At day 2 post-plating, cells were co-transfected with SIRT3 overexpression plasmid and siRNA LCAD, and 48 h afterwards cells were collected for analysis, as described in Section 2. Senescence staining protocol was performed 24 h before the last treatment. (**A**) qRT-PCR analysis of differentiation marker MAP2 and lipid accumulation marker PLIN2. *Hprt* was used as loading control. Data are expressed as fold change over control or siRNA LCAD groups (reference conditions, no tBHP added). (**B**) Representative images of SA-β-gal staining in NSCs exposed to tBHP treatment for 4 days (control group, no tBHP added), and subjected to SIRT3 overexpression, with or without additional LCAD silencing. H_2_O_2_ overnight treatment served as a positive control. Scale bar: 100 μm. Lower panels: selected sections enlarged 4×. (**C**) Quantitative analysis of cells positive for SA-β-gal for a given group, expressed as the percentage of the total number of cells. (**A**,**C**) Data represent mean values ± SEM for three independent experiments, yielding at least 7 data points per group. Each data point represents an individual value. ** *p* < 0.01 and *** *p* < 0.001 compared to control cells, ^§^ *p* < 0.05 and ^§§§^ *p* < 0.001 compared to tBHP-treated cells, ^‡^ *p* < 0.05 compared to SIRT3-transfected cells, and ^†^ *p* < 0.001 compared to SIRT3-transfected cells with or without tBHP treatment. Abbreviations: LCAD, long chain acyl-CoA dehydrogenase; MAP2, microtubule-associated protein 2; PLIN2, perilipin 2; SA-β-GAL, SA-β-galactosidase; siLCAD, LCAD silencing; tBHP, tert-butyl hydroperoxide.
