# Supplementary material for: The Mitochondrial Antioxidant Sirtuin3 Cooperates with Lipid Metabolism to Safeguard Neurogenesis in Aging and Depression
Source: Cells. 2021 Dec 29;11(1):90. doi: 10.3390/cells11010090 (PMC8750385; doi:10.3390/cells11010090)
Supplement: Supplementary file 1 [file cells-11-00090-s001.zip › cells-1471567-Supplementary.pdf]

cells

MDPI

Article: **The mitochondrial antioxidant Sirtuin3 cooperates with lipid metabolism to safeguard neurogenesis in ageing and depression**

Authors: **Sónia Sá Santos, João B. Moreira, Márcia Costa, Rui S. Rodrigues, Ana M. Sebastião, Sara Xapelli and Susana Solá**

Supplementary Figure

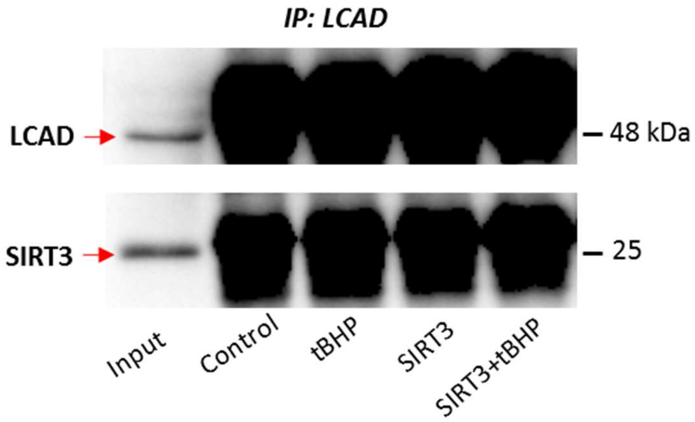

Figure S1. Detection of LCAD and SIRT3 proteins in input sample for the immunoprecipitation assays. Immunoblots correspond to Figure 3A for cell lysates immunoprecipitated with anti-LCAD antibody (lanes 2-5). Input sample (lane 1) corresponds to non-immunoprecipitated control group lysate. Abbreviations: IP, Immunoprecipitation; LCAD, long chain acyl-CoA dehydrogenase; tBHP, tert-butyl hydroperoxide.
